# LncRNA *ZNNT1* induces p53 degradation by interfering with the interaction between p53 and the SART3-USP15 complex

**DOI:** 10.1093/pnasnexus/pgad220

**Published:** 2023-07-04

**Authors:** Kenzui Taniue, Takeaki Oda, Tomoatsu Hayashi, Yuki Kamoshida, Yasuko Takeda, Anzu Sugawara, Yuki Shimoura, Lumi Negishi, Takeshi Nagashima, Mariko Okada-Hatakeyama, Yoshifumi Kawamura, Naoki Goshima, Nobuyoshi Akimitsu, Tetsu Akiyama

**Affiliations:** Laboratory of Molecular and Genetic Information, Institute for Quantitative Biosciences, The University of Tokyo, Tokyo 113-0032, Japan; Laboratory of Molecular and Genetic Information, Institute for Quantitative Biosciences, The University of Tokyo, Tokyo 113-0032, Japan; Laboratory of Molecular and Genetic Information, Institute for Quantitative Biosciences, The University of Tokyo, Tokyo 113-0032, Japan; Laboratory of Molecular and Genetic Information, Institute for Quantitative Biosciences, The University of Tokyo, Tokyo 113-0032, Japan; Laboratory of Molecular and Genetic Information, Institute for Quantitative Biosciences, The University of Tokyo, Tokyo 113-0032, Japan; Isotope Science Center, The University of Tokyo, Tokyo 113-0032, Japan; Isotope Science Center, The University of Tokyo, Tokyo 113-0032, Japan; Laboratory of Molecular and Genetic Information, Institute for Quantitative Biosciences, The University of Tokyo, Tokyo 113-0032, Japan; Cellular Systems Biology Team, RIKEN Genome Sciences Center (GSC), Kanagawa 230-0045, Japan; Present address: SCC Project Department, SRL Inc., Shizuoka 4111-8777, Japan; Cellular Systems Biology Team, RIKEN Genome Sciences Center (GSC), Kanagawa 230-0045, Japan; Institute for Protein Research, Osaka University, Osaka 565-0871, Japan; Research and Development Department, Fukushima Translational Research Foundation, Tokyo 103-0023, Japan; Department of Human Science, Musashino University, Tokyo 135-8181, Japan; Isotope Science Center, The University of Tokyo, Tokyo 113-0032, Japan; Laboratory of Molecular and Genetic Information, Institute for Quantitative Biosciences, The University of Tokyo, Tokyo 113-0032, Japan

**Keywords:** long noncoding RNA, *ZNNT1*, SART3, p53, tumorigenicity

## Abstract

Mammalian genomes encode large number of long noncoding RNAs (lncRNAs) that play key roles in various biological processes, including proliferation, differentiation, and stem cell pluripotency. Recent studies have addressed that some lncRNAs are dysregulated in human cancers and may play crucial roles in tumor development and progression. Here, we show that the lncRNA *ZNNT1* is required for the proliferation and tumorigenicity of colon cancer cells with wild-type p53. *ZNNT1* knockdown leads to decreased ubiquitination and stabilization of p53 protein. Moreover, we demonstrate that *ZNNT1* needs to interact with SART3 to destabilize p53 and to promote the proliferation and tumorigenicity of colon cancer cells. We further show that SART3 is associated with the ubiquitin-specific peptidase USP15 and that *ZNNT1* may induce p53 destabilization by inhibiting this interaction. These results suggest that *ZNNT1* interferes with the SART3-USP15 complex-mediated stabilization of p53 protein and thereby plays important roles in the proliferation and tumorigenicity of colon cancer cells. Our findings suggest that *ZNNT1* may be a promising molecular target for the therapy of colon cancer.

**Significance Statement**

It has been shown that the expression of many lncRNAs is dysregulated in several types of cancer, including colon cancer, and some lncRNAs have oncogenic or tumor-suppressive functions. Here, we show that the lncRNA *ZNNT1* is required for the proliferation and tumorigenicity of colon cancer cells with wild-type p53. We demonstrate that *ZNNT1* interacts with the multifunctional RNA-binding protein SART3 to destabilize p53. We further show that SART3 is associated with the ubiquitin-specific peptidase USP15 and that *ZNNT1* induces p53 destabilization by inhibiting the interaction between p53 and the SART3-USP15 complex. Our findings may provide critical insights into cancer therapy. In particular, *ZNNT1* may be a promising molecular target for the therapy of colon cancer.

## Introduction

Mammalian genomes encode numerous long noncoding RNAs (lncRNAs), which are defined as RNA genes larger than 200 bp that appear to have little or no coding potential (1, 2). Many lncRNAs are expressed in a developmentally regulated and cell type–dependent manner. Accumulating evidence indicates that lncRNAs play critical roles in diverse biological processes, including proliferation, differentiation, embryogenesis, neurogenesis, and stem cell pluripotency (3–6). lncRNAs have a number of functions such as chromatin and genomic structural remodeling, RNA stabilization, and transcriptional regulation (6). It has also been reported that lncRNAs regulate the stability of proteins by preventing posttranslational modifications associated with protein degradation (7). Furthermore, it has been shown that the expression of many lncRNAs is dysregulated in several types of cancer, including colon cancer, and some lncRNAs have oncogenic or tumor-suppressive functions (5, 7–11). However, the detailed mechanisms by which lncRNAs contribute to tumor development and progression remain to be elucidated.

The tumor suppressor p53 is well known for its role as a transcription factor (12). p53 is a cellular stress sensor that responds to signals such as DNA damage, oncogene expression, and hypoxia and causes cell cycle arrest, senescence, and/or apoptosis (13). Moreover, p53 regulates other biological processes, including autophagy, metabolic homeostasis, and stem cell pluripotency (13). Transactivation of specific target genes by p53 is believed to be critical for these p53 functions, although some p53 functions may be independent of transactivation (14). p53 is an unstable protein with an in vivo half-life of less than 20 min (15). Its stability is regulated by its ubiquitin-mediated proteasomal degradation (16). Mdm2 is known to be an important ubiquitin ligase (E3) that promotes p53 degradation and/or inactivation of its function (17). RNA molecules such as mRNA, miRNA, circRNA, and lncRNA have also been reported to regulate p53 expression and function (18, 19). Furthermore, RNA-binding proteins (RBPs), including hnRNPK and hnRNPL, have also been shown to be associated with p53 (20, 21), suggesting that p53 stability and/or function are modulated by RNAs.

Squamous cell carcinoma antigen recognized by T cells 3 (SART3)/HIV-1 Tat-interacting protein of 110 kDa (Tip110) is a nuclear RBP, which contains two RNA recognition motifs (RRM1 and RRM2) near its C-terminus (22). SART3 has been studied as a target antigen for immunotherapy (22). SART3 plays roles in stem cell proliferation and differentiation, and embryogenesis by regulating transcription, pre-mRNA splicing, spliceosome assembly, and mRNA synthesis (22). Moreover, SART3 is expressed at high levels in the nucleus of malignant tumor cell lines and a majority of cancer tissues as well as stem cells (23–27).

We previously established cell lines with different tumorigenicity and found that lncRNA *UPAT* plays critical roles in the colon tumorigenesis (7). In the present study, we show that the lncRNA *ZNNT1* is required for the tumorigenicity of colon cancer cells with wild-type p53. We further show that *ZNNT1* promotes proteasomal degradation of p53 by interfering with the interaction between p53 and the SART3-USP15 complex.

## Results

### *ZNNT1* is required for the tumorigenicity of colon cancer cells with wild-type p53

We have previously established the weakly tumorigenic colon cancer cell line CCSC#11 from the highly tumorigenic cell line CCSC#P (7). To identify lncRNAs critical for the tumorigenicity of colon cancer cells, we compared gene expression patterns between CCSC#P and CCSC#11 (Table S1). We found that three genes encoding lncRNAs were down-regulated in CCSC#11 cells (Tables S1 and S2), including *UCA1* (FR407590) and *ZNNT1* (NR_164368.1). We then examined the effects of siRNA-mediated knockdown of the three candidate genes on the proliferation of colon cancer HCT116 cells. CellTiter-Glo assays revealed that knockdown of either *UCA1* or *ZNNT1*, but not *CR621874*, caused a significant reduction in the growth of HCT116 cells (Fig. S1A–C). Consistent with these results, *UCA1* has previously been reported to be involved in the tumorigenicity of several cancers (28). Interestingly, in contrast to our results, *ZNNT1* has recently been reported to inhibit tumorigenesis of uveal melanoma by inducing autophagy (29). We therefore decided to focus our analysis on *ZNNT1*, which is expressed specifically in humans and is not conserved in other vertebrates (Fig. S1D).

**Fig. 1.**
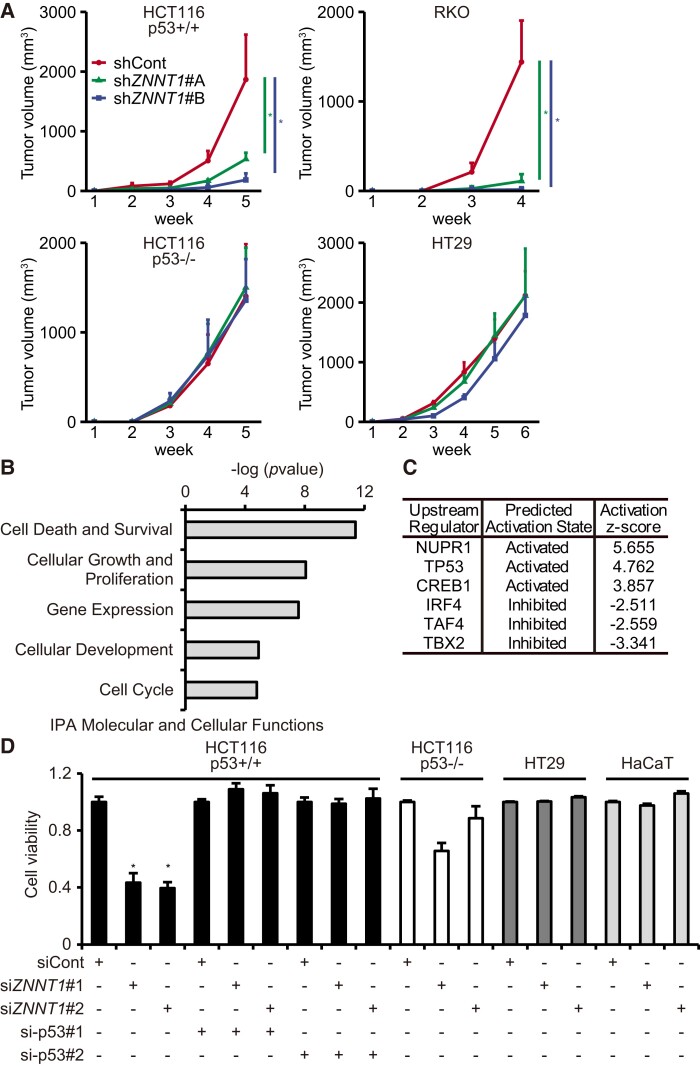
*ZNNT1* is required for the tumorigenicity of colon cancer cells with wild-type p53. A) HCT116 (p53^+/+^) (upper left), RKO (upper right), HCT116 (p53^−/−^) (lower left), and HT29 (lower right) cells infected with a lentivirus expressing an shRNA targeting *ZNNT1* were transplanted into nude mice. Results are expressed as the mean ± SD (*n* = 6). *P < 0.05. B) IPA “Molecular and Cellular Functions” analysis of *ZNNT1*-regulated genes. C) The results of IPA Upstream Regulator analyses of *ZNNT1*-regulated genes. D) Viability of HCT116 (p53^+/+^), HCT116 (p53^−/−^), HT29, and HaCaT cells transfected with a siRNA targeting *ZNNT1* along with siCont or si-p53 was assessed by CellTiter-Glo assays. Results are expressed as the mean ± SD (*n* = 4). *P < 0.05.

To clarify the significance of *ZNNT1* in colon tumorigenesis, we infected the colon cancer cell line HCT116 with a lentivirus expressing an shRNA targeting *ZNNT1* (sh*ZNNT1*). When transplanted into nude mice, the growth of HCT116 cells was significantly retarded compared with cells infected with a control lentivirus (Figs. 1A and S1E). Thus, *ZNNT1* may play an essential role in the tumorigenicity of colon cancer cells. We next examined *ZNNT1* expression in human colon tumor and adjacent noncancerous tissues by qRT–PCR analysis. We found that *ZNNT1* expression was higher in colon tumors than in adjacent noncancerous tissues (Fig. S1F). In addition, subcellular fractionation and qRT–PCR analysis revealed that *ZNNT1* was present in the nucleus (Fig. S1G).

To elucidate the potential function of *ZNNT1* in colon cancer cells, we analyzed *ZNNT1*-regulated genes in HCT116 cells (Fig. S1H). RNA-seq analysis showed that knockdown of *ZNNT1* using siRNA resulted in the up-regulation of 1,201 genes and down-regulation of 905 genes (Tables S3 and S4). Functional pathway analysis using the Ingenuity Pathway Analysis (IPA) software revealed that the gene expression signatures observed in *ZNNT1*-knockdown cells were enriched for those involved in “Cell Death and Survival” and “Cellular Growth and Proliferation” (Fig. 1B and Tables S3 and S4). Moreover, we found a signature that overlaps with that regulated by the transcription factor p53 (Fig. 1C and Table S5). We therefore investigated the role of p53 in the function of *ZNNT1* in HCT116 cells, which contains wild-type p53: we examined the effect of *ZNNT1* knockdown on the tumorigenicity of HCT116 (p53^−/−^), a derivative of HCT116, in which p53 was disrupted by homologous recombination (30). We observed that knockdown of *ZNNT1* did not show any effect on the tumorigenicity of HCT116 (p53^−/−^) cells (Fig. 1A). Consistent with these results, *ZNNT1* knockdown inhibited the tumorigenicity of RKO cells, which contain wild-type p53, but not of HT29 cells, which contain mutated p53 (Fig. 1A).

We next examined the effects of siRNA-mediated knockdown of either *ZNNT1* or p53 on the proliferation of colon cancer cells. Consistent with the above results, CellTiter-Glo assays revealed that knockdown of *ZNNT1* by siRNA caused a significant reduction in the growth of HCT116 (p53^+/+^) cells, but not of HCT116 (p53^−/−^) cells or HT29 cells, which contain mutated p53 (Figs. 1D and S1C and I). Furthermore, we observed that si*ZNNT1*-mediated growth reduction was suppressed in cells transfected with a siRNA targeting p53 (Figs. 1D and S1C and J). We also found that knockdown of *ZNNT1* resulted in marked increases in apoptotic death of HCT116 cells, as determined by annexin assays (Fig. S1K). In addition, *ZNNT1* knockdown did not affect the proliferation of normal keratinocyte HaCaT cells in vitro (Fig. 1D). On the other hand, *ZNNT1* overexpression as well as p53 knockdown did not influence the proliferation of HCT116 (p53^+/+^) cells (Fig. S1L and M).

Taken together, these results suggest that *ZNNT1* is required for the escape of colon tumor cells from p53-mediated inhibition of the proliferation and tumorigenicity.

### *ZNNT1* knockdown leads to the stabilization of p53 protein, but not mRNA

**Fig. 2.**
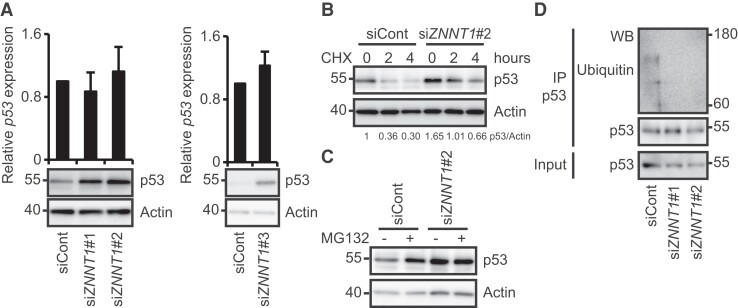
Knockdown of *ZNNT1* inhibits the degradation and ubiquitination of p53 protein in colon cancer cells. A) (Upper) qRT–PCR analysis of *p53* expression in HCT116 cells transfected with siRNA targeting *ZNNT1*. Results are expressed as the mean ± SD (*n* = 3). (Lower) Cell lysates were subjected to immunoblotting analysis with anti-p53 or anti-actin antibody. Actin was used as a negative control. B) HCT116 cells transfected with a siRNA targeting *ZNNT1* were treated with CHX for the indicated times and then subjected to immunoblotting analysis with anti-p53 or anti-actin antibody. Actin was used as a negative control. C) HCT116 cells transfected with a siRNA targeting *ZNNT1* were cultured in the presence or absence of MG132 and then subjected to immunoblotting analysis with anti-p53 or anti-actin antibody. Actin was used as a negative control. D) Lysates from HCT116 cells that had been transfected with siRNA targeting *ZNNT1* and treated with MG132 were subjected to immunoprecipitation with anti-p53 antibody followed by immunoblotting analysis with anti-ubiquitin or anti-p53 antibody.

It has been reported that the stability of p53 is regulated by proteasome-mediated degradation (15). We therefore investigated whether *ZNNT1* is involved in the regulation of p53 expression in HCT116 cells. qRT–PCR and immunoblotting analyses revealed that knockdown of *ZNNT1* using siRNA resulted in a marked increase in the levels of p53 protein, but not of mRNA (Fig. 2A). Consistent with these results, *ZNNT1* knockdown led to the up-regulation of p21, a target gene of p53, in HCT116 cell (Fig. S2). We found that knockdown of *ZNNT1* enhanced the stability of p53 protein in HCT116 cells treated with cycloheximide (CHX) (Fig. 2B). We also observed that treatment of cells with the proteasome inhibitor MG132 inhibited the increase in the p53 protein levels caused by knockdown of *ZNNT1* (Fig. 2C). Moreover, we found that overexpression of *ZNNT1* decreased the levels of p53 protein and this reduction was restored by treatment with MG132 (Fig. S2B). Furthermore, we found that *ZNNT1* knockdown resulted in decreased ubiquitination of p53 (Fig. 2D). These results suggest that *ZNNT1* destabilizes p53 protein by promoting its ubiquitination and proteasome-mediated degradation in colon cancer cells.

### *ZNNT1* is associated with SART3 in colon cancer cells

**Fig. 3.**
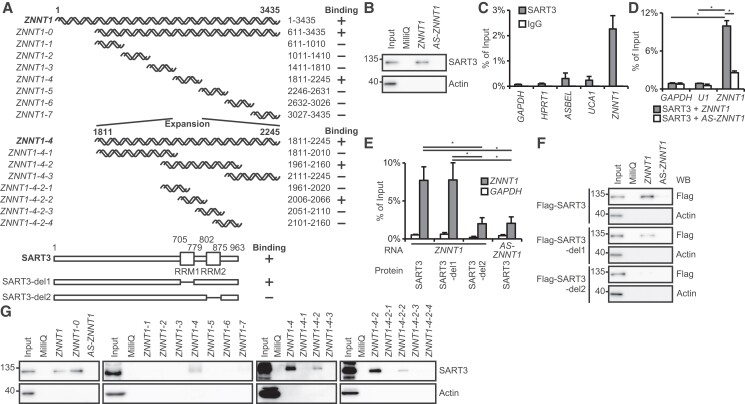
*ZNNT1* is associated with SART3 in colon cancer cells. A) Schematic representation of the *ZNNT1* (upper) and SART3 protein (lower). Mutants used in RIP (E) and pull-down (F, G) assays are also shown. B) Biotinylated sense or antisense *ZNNT1* generated in vitro was incubated with lysates from HCT116 cells and was then precipitated with streptavidin beads followed by immunoblotting analysis with anti-SART3 or anti-actin antibody. *AS-ZNNT1*, in vitro-transcribed antisense *ZNNT1*. Actin was used as a negative control. C) Lysates from HCT116 cells were subjected to immunoprecipitation with anti-SART3 antibody or rabbit IgG followed by qRT–PCR analysis to detect *ZNNT1* mRNA. *GAPDH* mRNA, *HPRT1* mRNA, *ASBEL*, and *UCA1* were used as negative controls. Results are expressed as the mean ± SD (*n* = 2). See also Fig. S3B. D) Lysates from HCT116 cells transfected with sense (*ZNNT1*) or antisense *ZNNT1* (*AS-ZNNT1*) and Flag-SART3 were subjected to immunoprecipitation with anti-Flag antibody followed by qRT–PCR analysis to detect *ZNNT1* mRNA. *AS-ZNNT1*, *GAPDH* mRNA, and *U1* small nuclear RNA were used as negative controls. Results are expressed as the mean ± SD (*n* = 3). E) Lysates from HCT116 cells transfected with *ZNNT1* along with wild-type or mutant Flag-SART3 were subjected to immunoprecipitation with anti-Flag antibody followed by qRT–PCR analysis to detect *ZNNT1* and *GAPDH* mRNA. Results are expressed as the mean ± SD (*n* = 3). F) Biotinylated sense (*ZNNT1*) or antisense *ZNNT1* (*AS-ZNNT1*) generated in vitro was incubated with lysates from HCT116 cells transfected with wild-type or mutant Flag-SART3 and was precipitated with streptavidin beads followed by immunoblotting analysis with anti-Flag or anti-actin antibody. Actin was used as a negative control. G) Biotinylated sense or mutant *ZNNT1* generated in vitro was incubated with lysates from HCT116 cells and was precipitated with streptavidin beads followed by immunoblotting analysis with anti-SART3 or anti-actin antibody. Actin was used as a negative control.

Many lncRNAs have been shown to exert their biological functions by forming complexes with proteins (1, 31). We performed RNA pull-down assays to identify proteins that could associate with *ZNNT1*. Proteins that coprecipitated with in vitro synthesized *ZNNT1-0* were separated by sodium dodecyl sulfate–polyacrylamide gel electrophoresis (SDS–PAGE). A band that specifically coprecipitated with sense *ZNNT1-0*, but not with antisense *ZNNT1-0*, was excised and subjected to liquid chromatograph–mass spectrometry (LC–MS/MS) analysis (Figs. 3A and S3A). Among eight protein candidates identified, the peptides derived from the multifunctional RBP SART3 (22) were most frequently detected (Table S6). Consistent with this, RNA pull-down assays revealed that SART3 precipitated with *ZNNT1* generated in vitro, but not with antisense *ZNNT1* (Fig. 3A and B). We also performed RNA immunoprecipitation (RIP) assays with anti-SART3 antibody using lysates from HCT116 cells. qRT–PCR analysis of the immunoprecipitates revealed that SART3 was associated with endogenous *ZNNT1*, but not with *GAPDH* mRNA, *HPRT1* mRNA, *UCA1*, or *ASBEL* RNA (8, 9) (Fig. 3C). In a parallel experiment, we confirmed that SART3 was also associated with the small nuclear RNA *U6*, but not with *U1* as reported previously (32) (Fig. S3B). In addition, RIP analysis with anti-Flag antibody using lysates from HCT116 cells transfected with Flag-tagged SART3 revealed that exogenously expressed SART3 was also associated with *ZNNT1*, but not with antisense *ZNNT1*, *GAPDH* mRNA, or *U1* small nuclear RNA (Fig. 3D).

RIP assays using a series of SART3 deletion mutants revealed that the region from amino acids 802 to 875 (RRM2 in Fig. 3A) was required for the association of SART3 with *ZNNT1* (Fig. 3A and E). RNA pull-down assays using a series of SART3 deletion mutants and in vitro synthesized sense or antisense *ZNNT1* also showed that SART3 interacted with *ZNNT1*, but not with antisense *ZNNT1* and that RRM2 is required for this interaction (Fig. 3A and F). We also found that SART3 bound to the region from nucleotide 2006 to 2066 of *ZNNT1*, which contains the AAAGAG sequence that resembles the hexanucleotide, ACAGAG, reported to interact with SART3 (33) (Figs. 3A and G and S3C). These results suggest that *ZNNT1* is associated with SART3 in colon cancer cells.

### *ZNNT1* needs to bind to SART3 to destabilize p53

**Fig. 4.**
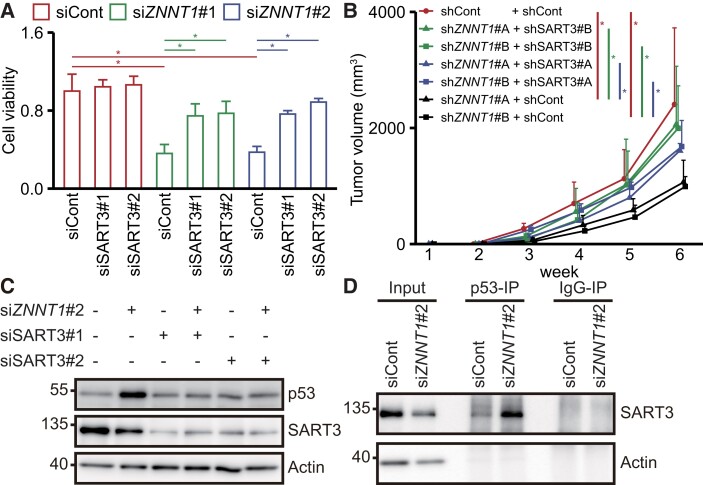
*ZNNT1* regulates the stabilization of p53 by interfering with the binding of SART3 to p53. A) Viability of HCT116 (p53^+/+^) cells transfected with siRNA targeting *ZNNT1* along with siCont or siSART3 was assessed by CellTiter-Glo assays. Results are expressed as the mean ± SD (*n* = 4). *P < 0.05. B) HCT116 (p53^+/+^) cells infected with a lentivirus expressing a shRNA targeting *ZNNT1* along with SART3 were transplanted into nude mice. Results are expressed as the mean ± SD (*n* = 6). *P < 0.05. C) Lysates from HCT116 cells transfected with siRNA targeting *ZNNT1* and/or SART3 were subjected to immunoblotting analysis with anti-p53, anti-SART3, and anti-actin antibodies. Actin was used as a negative control. D) Lysates from HCT116 cells transfected with siZNNT1 were subjected to immunoblotting analysis with anti-p53 antibody or mouse IgG followed by immunoblotting analysis with anti-SART3 and anti-actin antibodies. Actin was used as a negative control.

We next examined whether SART3 is involved in the *ZNNT1*-mediated regulation of proliferation and tumorigenicity of colon cancer cells. Knockdown of SART3 partially rescued HCT116 cells from the reduction in cell viability caused by *ZNNT1* knockdown (Figs. 4A and S4A). Furthermore, SART3 knockdown restored the tumorigenicity of HCT116 cells infected with a lentivirus targeting *ZNNT1* (Figs. 4B and S4B). Consistent with these results, *ZNNT1* knockdown barely induced p53 up-regulation when SART3 was also knocked down (Fig. 4C). In contrast, we found that SART3 knockdown had no effect on either *ZNNT1* or p53 expression in HCT116 cells (Figs. 4C and S4A). Knockdown of *ZNNT1* also did not affect the expression level of SART3 in HCT116 cells (Figs. 4C and S4C). Furthermore, *ZNNT1* knockdown resulted in a significant increase in SART3 association with p53 in HCT116 cells (Figs. 4D and S4D). In addition, we found that p53 protein levels were decreased by overexpression of *ZNNT1*, but not of *ZNNT1-1*, a mutant *ZNNT1* lacking the SART3-binding region (Fig. S4E). Taken together, these results raise the possibility that *ZNNT1* interferes with SART3-mediated stabilization of p53 and is required for the tumorigenicity of colon cancer cells.

### *ZNNT1* interferes with the SART3-USP15 complex-mediated stabilization of p53

**Fig. 5.**
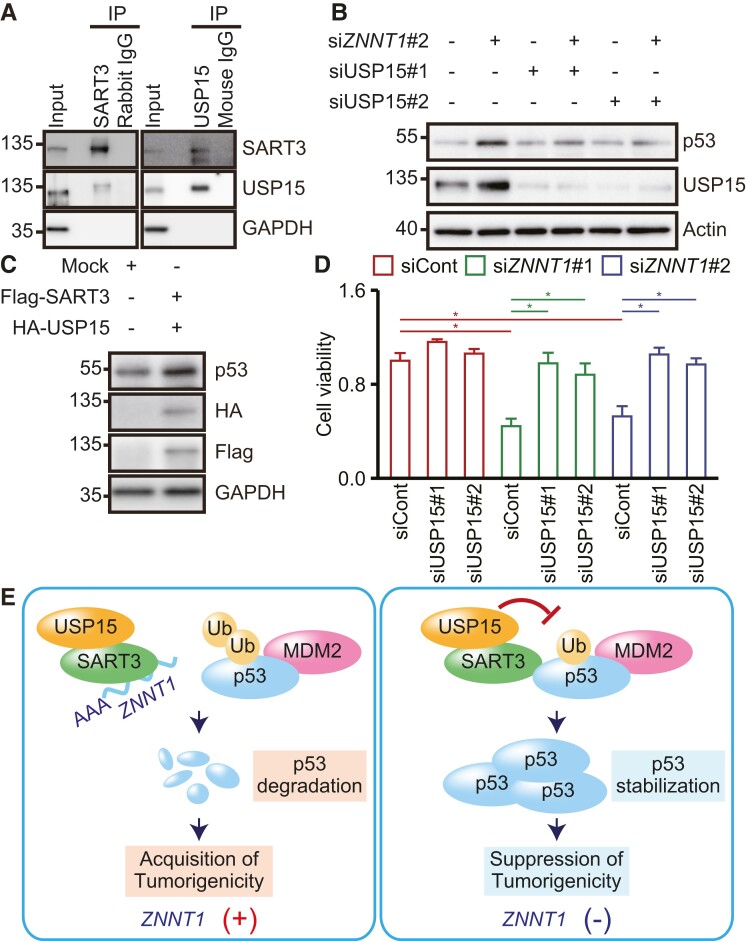
*ZNNT1* regulates stabilization of p53 by interfering with its binding to the SART3-USP15 complex. A) USP15 is associated with SART3 in vivo. Lysates from HCT116 cells were subjected to immunoprecipitation with anti-SART3 antibody, anti-USP15 antibody, rabbit IgG, or mouse IgG followed by immunoblotting analysis with anti-SART3, anti-USP15, or anti-GAPDH antibody. GAPDH was used as a negative control. B) Lysates from HCT116 cells transfected with siRNA targeting *ZNNT1* and/or USP15 were subjected to immunoblotting analysis with anti-p53, anti-USP15, or anti-actin antibody. Actin was used as a negative control. C) Lysates from HCT116 cells transfected with HA-tagged USP15 and Flag-tagged SART3 were subjected to immunoblotting analysis with anti-p53, anti-HA, anti-Flag, or anti-GAPDH antibody. GAPDH was used as a negative control. D) Viability of HCT116 (p53^+/+^) cells transfected with siRNA targeting *ZNNT1* along with siCont or siUSP15 was assessed by CellTiter-Glo assays. Results are expressed as the mean ± SD (*n* = 4). *P < 0.05. E) A schematic model showing the mechanism of p53 regulation by *ZNNT1* and SART3-USP15 complex. *ZNNT1* interacts with SART3 to interfere with its association with p53 and thereby facilitates tumorigenesis.

To identify factors involved in SART3-mediated stabilization of p53, we immunoprecipitated SART3 from HCT116 cell lysates and analyzed coprecipitated proteins by LC–MS/MS (Fig. S5A and Table S7). In agreement with recent reports (34, 35), we identified peptides derived from the ubiquitin-specific peptidase USP15, in addition to peptides derived from known SART3-binding proteins, including USP4 (36) and PRPF31 (35) (Table S7). Pull-down assays using either anti-SART3 or anti-USP15 antibody confirmed the association of SART3 with USP15 in HCT116 cells (Fig. 5A). It has been reported that SART3 enhances USP15 binding to ubH2B and facilitates deubiquitination of ubH2B in free histones (34). We therefore hypothesized that SART3 recruits USP15 to p53 and stabilizes p53 in *ZNNT1*-knockdown cells. Consistent with this notion, knockdown of *ZNNT1* barely up-regulated the p53 protein when USP15 was also knocked down (Figs. 5B and S5B). In contrast, we found that USP15 knockdown alone had no effect on p53 expression in HCT116 cells (Figs. 5B and S5B). We also found that coexpression of USP15 and SART3 up-regulated the p53 protein in HCT116 cells (Fig. 5C). Moreover, USP15 knockdown partially rescued HCT116 cells from the reduction in cell viability caused by *ZNNT1* knockdown (Fig. 5D). Furthermore, RIP assays with HCT116 cell lysates using anti-USP15 antibody revealed that USP15 was associated with endogenous *ZNNT1*, but not with *GAPDH* mRNA, *HPRT1* mRNA, *U1* snRNA, *UCA1*, or *ASBEL* RNA (Fig. S5C). These results suggest that *ZNNT1* interacts with the SART3-USP15 complex and interferes with its binding to p53, and thereby induces p53 degradation (Fig. 5E).

## Discussion

In this study, we attempted to identify lncRNAs that are critical for colon tumorigenesis. We found that the lncRNA *ZNNT1* is overexpressed in colon cancers compared with adjacent noncancerous tissues and its knockdown inhibits the proliferation and tumorigenicity of colon cancer cells with wild-type but not mutant p53. Furthermore, we observed that *ZNNT1* destabilizes p53 protein by promoting its ubiquitination and proteasome-mediated degradation in colon cancer cells. Our findings suggest that *ZNNT1* promotes colon cancer cell proliferation and tumorigenesis by inducing p53 destabilization.

*ZNNT1*, which is not conserved across vertebrates, has been reported to inhibit the tumorigenesis and migration of uveal melanoma cells by inducing autophagy (29). Thus, *ZNNT1* functions differently in uveal melanoma and colon cancer: *ZNNT1* may act in a tumor-suppressive manner in uveal melanoma cells but in an oncogenic manner in colon cancer cells. In our future studies, we would like to clarify the mechanisms by which *ZNNT1* exhibits different functions in uveal melanoma cells and colon cancer cells.

To elucidate the mechanism by which *ZNNT1* destabilizes p53, we searched for *ZNNT1*-associated proteins and found that *ZNNT1* interacts with SART3. Although SART3 has been known to function as a recycling factor of the RNA splicing machinery (22) we found that *ZNNT1* interacts with SART3 to regulate p53 stabilit*y*. We observed that *ZNNT1* knockdown markedly increases the association of SART3 with p53. We also found that *ZNNT1* knockdown does not induce p53 up-regulation when SART3 is also knocked down. These results imply that SART3 interacts with p53 and induces its up-regulation and that *ZNNT1* inhibits this interaction and p53 up-regulation. Furthermore, we observed that knockdown of SART3 restores *ZNNT1* knockdown-mediated reduction in the proliferation and tumorigenicity of colon cancer cells. Taken together, these results suggest that *ZNNT1* inhibits SART3-mediated p53 stabilization and thereby promotes the proliferation and tumorigenicity of colon cancer cells.

It is well known that p53 stability is regulated by its ubiquitination and deubiquitination. Consistent with this notion, we found that SART3 is associated with the deubiquitinase USP15 in colon cancer cells. We speculated that SART3 recruits USP15 to p53 and stabilizes p53 in *ZNNT1*-knockdown cells. Indeed, we observed that *ZNNT1* knockdown barely affects p53 stability when USP15 is also knocked down. We also found that USP15 knockdown alone has no effect on p53 expression. Furthermore, we observed that USP15 knockdown partially rescues HCT116 cells from *ZNNT1* knockdown-induced reduction in cell viability. Thus, *ZNNT1* may inhibit the interaction between the SART3-USP15 complex and p53, and thereby induces p53 degradation and cell proliferation. However, it remains to be elucidated how the SART3-USP15 complex affects p53 ubiquitination by MDM2 and other ubiquitin ligases.

In our previous study, we established cell lines with different tumorigenicity and performed RNA-seq analysis to identify lncRNA *UPAT*, which plays a critical role in the proliferation and tumorigenicity of colon cancer cells. In the present study, we modified the annotation list used for RNA-seq analysis, which led to the discovery of *ZNNT1*. In addition to *ZNNT1*, we found that two additional lncRNAs, including *UCA1*, are also up-regulated in the cell lines with high tumorigenicity. *UCA1* has already been reported to be involved in the tumorigenicity of several types of cancer. However, we found that knockdown of *CR621874* does not cause a significant reduction in the growth of colon cancer cells.

In conclusion, we have shown that *ZNNT1* plays a critical role in the tumorigenicity of colon cancer cells with wild-type p53. We further showed that *ZNNT1* induces p53 degradation by interfering with the interaction between p53 and the SART3-USP15 complex (Fig. 5D). Our findings may provide critical insights into cancer therapy.

## Materials and methods

Detailed materials and methods are provided in the Supplementary material. The sequences of shRNAs, siRNAs, and primers are listed in Tables S8–S10.

### CellTiter-Glo assay

Cell viability was determined by measuring the intracellular levels of adenosine triphosphate (ATP) using the CellTiter-Glo Luminescent Cell Viability Assay kit (Promega). Luminescence was measured using a Mithras LB 940 (Berthold) or a GloMax Discover Microplate Reader (Promega).

### RNA pull-down assay

Biotinylated *ZNNT1*, antisense-*ZNNT1*, or its deletion mutants were incubated with lysates (200 μg) from HCT116 cells and then mixed with streptavidin beads, washed, and boiled in SDS buffer as described previously (7). The associated proteins were resolved by gel electrophoresis and visualized by silver staining or immunoblotting. Specific bands were excised and identified by an automated LC–MS/MS system, which consists of the Zaplous Advance nano UHPLC HTS-PAL xt system (AMR) equipped with a Zaplous α Pep-C18 packed column (3 μm, 0.1 × 150 mm) (AMR) and an LTQ Velos Orbitrap ETD instrument (Thermo Fischer Scientific) as described previously (37). For protein identification, spectra were processed using Proteome Discoverer Version 1.4 (Thermo Fisher Scientific) against SEQUEST and subjected to a 5% false discovery rate (FDR) cutoff.

## Supplementary Material

Supplementary material is available at *PNAS Nexus* online.

## Supplementary Material

pgad220_Supplementary_Data

## Data Availability

All study data are included in the article and/or Supplementary material. The RNA-seq data generated in this study have been deposited in the DNA Data Bank of Japan Sequence Read Archive (DRA) database (accession number: DRA014973).
