# Supplementary material for: LncRNA ZNNT1 induces p53 degradation by interfering with the interaction between p53 and the SART3-USP15 complex
Source: PNAS Nexus. 2023 Jul 4;2(7):pgad220. doi: 10.1093/pnasnexus/pgad220 (PMC10337854; doi:10.1093/pnasnexus/pgad220)
Supplement: pgad220_Supplementary_Data [file pgad220_supplementary_data.zip › PNASNEXUS-PNASNEXUS-2023-00290R-s11.docx]

**
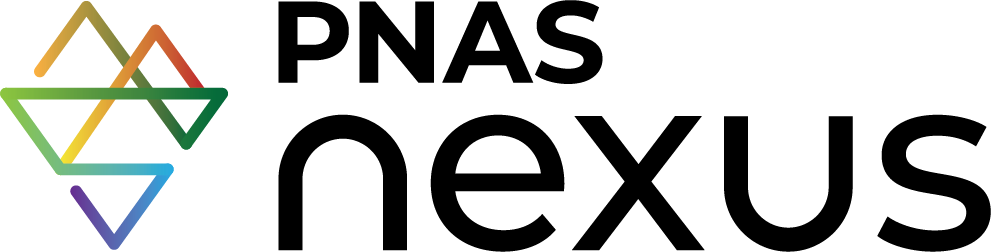
**

**Supplementary Information for**

LncRNA *ZNNT1* induces p53 degradation by interfering with the interaction between p53 and the SART3-USP15 complex

Kenzui Taniue^a^, Takeaki Oda^a^, Tomoatsu Hayashi^a^, Yuki Kamoshida^a^, Yasuko Takeda^a^, Anzu Sugawara^b^, Yuki Shimoura^b^, Lumi Negishi^a^, Takeshi Nagashima^c,1^, Mariko Okada-Hatakeyama^c,d^, Yoshifumi Kawamura^e^, Naoki Goshima^f^, Nobuyoshi Akimitsu^b^ and Tetsu Akiyama^a,*^

Tetsu Akiyama

Email: [akiyama@iqb.u-tokyo.ac.jp](mailto:akiyama@iqb.u-tokyo.ac.jp)

**This PDF file includes:**

SI Materials and Methods

SI References

Figures S1 to S5

**Other supplementary materials for this manuscript include the following:**

Dataset - "Tables S1-S10"

**SI Materials and Methods**

**Cell culture**

CCSC#P cells were purchased from Celprogen (San Pedro, CA, Human colon cancer stem cell, Cat. #36112-39, Lot. #710011-05). CCSC#P and CCSC#11 cells were cultured in DMEM/F12 supplemented with 10% bovine serum. HCT116(p53+/+) and HCT116(p53-/-), a generous gift from Prof. B. Vogelstein, Johns Hopkins University, and HT29 (ATCC) cells were cultured in McCoy's 5A supplemented with 10% bovine serum. RKO cells (ATCC) were cultured in MEM supplemented with 10% bovine serum. HaCaT and 293FT cells (ATCC) were cultured in DMEM supplemented with 10% bovine serum.

**Antibodies and regents**

Anti-FLAG (F3165) and anti-actin (A-2066) antibodies were obtained from Sigma. Anti-p53 (DO-1) antibody was obtained from Santa Cruz Biotechnology. Anti-SART3 (A301-521A) antibody was obtained from Bethyl Laboratories. Anti-USP15 (1C10) antibody was obtained from Abnova. Anti-GAPDH (MAB374) antibody was obtained from Merck Millipore. Anti-Ubiquitin (3936) antibody was obtained from Cell Signaling. Secondary antibodies and ECL-plus were purchased from GE Healthcare. Cycloheximide and MG132 were purchased from Sigma and Peptide Institute, respectively.

**Lentivirus production**

Lentiviral vector (CS-Rfa-CG) harboring an shRNA driven by the H1 promoter was transfected with the packaging vectors pCAG-HIV-gp and pCMV-VSV-G-RSV-Rev into 293FT cells using polyethylenimine 'MAX' (PEI, Polyscience, Inc. Cat. 24765). All plasmids were kindly provided by H. Miyoshi (RIKEN BioResource Center, Japan). Virus supernatants were purified by ultracentrifugation at 25,000 rpm for 90 min (SW28 rotor, Beckman). Infection efficiency was monitored by GFP expression as it is driven by the CMV promoter. The sequences of shRNAs are shown in Table S8.

**Tumorigenesis assay**

HCT116(p53+/+), HCT116(p53-/-), RKO and HT29 cells infected with a lentivirus expressing an shRNA targeting *ZNNT1* and/or SART3 were injected subcutaneously into 6-week-old nude mice (BALB/cAJcl-*nu*/*nu*, CLEA Japan). All animal experimental protocols were performed in accordance with the guidelines of the Animal Ethics Committee of the University of Tokyo.

**RNA interference**

siRNA duplexes targeting *ZNNT1* were purchased from Cosmo Bio and GeneDesign. Stealth siRNA duplexes targeting TP53 were purchased from Invitrogen. siRNA duplexes targeting SART3 were purchased from Ambion. Cells were transfected with RNA duplexes using Lipofectamine RNAiMAX (Invitrogen). Sequences of siRNAs are shown in Table S8. Validated Stealth negative control RNAi duplex with MED GC content #2 (Invitrogen), or Silencer Select negative control siRNA #2 (Ambion) was used as a control.

**qRT-PCR analysis**

Total RNA was isolated using the Total RNA Isolation kit (MACHEREY-NAGEL) and treated with DNase I (TAKARA). One microgram RNA was reverse transcribed using PrimeScript RT Master Mix (TAKARA, RR036A). qRT-PCR analysis of cDNA was performed on a LightCycler 480 (Roche Applied Science) using Syber Green PCR mastermix (Applied Biosystems). TissueScan Cancer and Normal Tissue cDNA Arrays (Colon Cancer cDNA Array III) were obtained from OriGene Technologies. Prior to fold-change calculation, the values were normalized to the signal generated from *GAPDH* or *β-actin* mRNA. Primer sequences are listed in Table S9.

**Constructs and transfection**

*ZNNT1*, SART3 and USP15 were amplified by PCR and cloned into pcDNA3.1(+), pcDNA3.1(+)-Flag, or pcDNA3.1(+)-HA, respectively. For pull-down assays, *ZNNT1* and its deletion mutants were cloned into pcDNA3.1(+) or pBlueScript II SK+. Primer sequences are listed in Table S10. Plasmids were transfected into cells using polyethylenimine 'MAX' (PEI, Polyscience, Inc. Cat. 24765).

**Apoptosis**

Cells were seeded in a 24-well plate at 24 h before transfection and then transfected with siRNA targeting *ZNNT1*. At 96 h after transfection, cells were washed with PBS and collected into a 15 mL tube. Phosphatidylserine exposure at the cell surface was detected using the MEBCYTO Apoptosis Kit (4700, MBL) according to the manufacturer’s protocol. The percentage of Annexin(+)/PI(−) cells and DNA content were measured by flow cytometry (FACSAria Cell Sorter; Becton Dickinson).

**Subcellular fractionation**

Cell pellets were resuspended in 1 packed cell volume of Hypotonic buffer (10 mM HEPES pH 7.5, 10 mM KCl, 1.5 mM MgCl_2_, 0.5% NP40). After incubation on ice for 10 min, cells were disrupted by 10 passages through a 25-gauge needle. Cells were centrifuged for 10 min at 1,000 g at 4°C and the supernatant containing the cytoplasmic fraction was collected by further centrifugation at 15,000 g for 15 min. The remaining pellets were washed twice with Hypotonic buffer, resuspended in Hypertonic buffer (20 mM HEPES pH 7.5, 420 mM KCl, 1.5 mM MgCl_2_, 0.5% NP40) and incubated at 4°C for 30 min with gentle rotation. The supernatant containing the nuclear fraction was collected by centrifugation at 15,000 g for 15 min.

**Purification of SART3-binding proteins**

HCT116 cells were transfected with Flag-tagged SART3. After fixed with 1% formaldehyde for 10 min at 25°C, cells were lysed in Lysis buffer (50 mM HEPES pH7.5, 150 mM KCl, 0.5% NP40, 2 mM EDTA, 1 mM NaF) containing protease inhibitors and RNase Inhibitor (Promega). Cells were centrifuged at 15,000 r.p.m. for 20 min at 4°C and the supernatants were incubated with anti-FLAG M2 Magnetic Beads (SIGMA) for 2 h at 4°C with gentle rotation. The beads were washed twice with Wash buffer (1% Triton X-100, 50 mM HEPES pH7.5, 150 mM NaCl, 5 mM EDTA, 0.05% SDS) containing protease inhibitors and RNase Inhibitor (Promega), and then twice with PBS containing protease inhibitors and RNase Inhibitor (Promega). After washing, proteins were eluted by competition with FLAG peptide (Sigma) containing protease inhibitors. Eluted proteins were desalted by methanol–chloroform precipitation, digested with trypsin (Promega) and then loaded on an automated LC-MS/MS system, which consists of the Zaplous Advance nano UHPLC HTS-PAL xt system (AMR) equipped with a Zaplous α Pep-C18 packed column (3 μm, 0.1 x 150 mm) (AMR) and an LTQ Velos Orbitrap ETD instrument (Thermo Fischer Scientific) as described previously (1). For protein identification, spectra were processed using Proteome Discoverer Version 1.4 (Thermo Fisher Scientific) against SEQUEST and subjected to a 5% false discovery rate (FDR) cutoff.

**RIP assay**

RIP assays was performed as described previously (2, 3) with minor modifications. Cells growing in 6-well dishes were lysed in 0.5 ml of Lysis buffer (50 mM HEPES pH7.5, 150 mM KCl, 0.5% NP40, 2 mM EDTA, 1 mM NaF) containing protease inhibitors and RNase Inhibitor (Promega), and centrifuged at 13,000 r.p.m. for 10 min. The supernatants were incubated with anti-SART3, anti-Flag, anti-mouse IgG or anti-rabbit IgG antibody for 3 h at 4°C with gentle rotation. Thirty microliters of Protein G Dynabeads (Invitrogen) were added and incubated for 1 h at 4°C with gentle rotation. The beads were washed thrice with Wash buffer (50 mM HEPES pH7.5, 150 mM KCl, 0.05% NP40) containing RNase Inhibitor (Promega) and then twice with PBS containing RNase Inhibitor (Promega). RNA was extracted using the Total RNA Isolation kit (MACHEREY-NAGEL) and qRT-PCR was performed as described above. Primer sequences for qRT-PCR are shown in Table S9.

**Immunoblotting**

Cells (5 x 10^6^) were lysed for 20 min with Lysis buffer (50 mM HEPES pH7.5, 150 mM KCl, 0.5% NP40, 2 mM EDTA, 1 mM NaF) containing protease inhibitors. After centrifugation at 13,000 r.p.m. for 20 min at 4°C, samples were resolved by SDS-PAGE, transferred to PVDF membranes (Immobilon-P, Millipore) and analyzed by immunoblotting using HRP-conjugated secondary antibodies. Membranes were blocked with 5% skimmed milk in TBS plus Tween 20 for 1 hour at 25°C before probing with antibodies. Visualization was performed using the Enhanced Chemiluminescence Plus Western Blotting Detection System (GE Healthcare) and LAS-4000EPUVmini Luminescent Image Analyzer (GE Healthcare).

**Immunoprecipitation**

Indicated expression plasmids were transfected into HCT116 cells that had been treated with MG132. Cells were lysed in Lysis buffer (50 mM HEPES pH7.5, 150 mM KCl, 0.5% NP40, 2 mM EDTA, 1 mM NaF) containing protease inhibitors and RNase Inhibitor (Promega), and centrifuged at 13,000 r.p.m. for 30 min. The supernatants were incubated with antibodies for 3 h at 4°C with gentle rotation. Thirty microliters of Protein G Dynabeads (Invitrogen) were added and incubated for 1 h at 4°C with gentle rotation. The beads were washed thrice with Wash buffer (50 mM HEPES pH7.5, 150 mM KCl, 0.05% NP40) containing protease inhibitors and RNase Inhibitor (Promega) and then twice with PBS containing protease inhibitors and RNase Inhibitor (Promega). After washing, immunocomplexes were analyzed by SDS-PAGE and immunoblotting with antibodies against target proteins.

***In vivo* ubiquitination assay**

HCT116 cells were transfected with siRNA targeting *ZNNT1*. After treatment with 10 μM MG132 for 3 h, cells were lysed in RIPA buffer (1% Nonidet-P40 (NP-40), 1% sodium deoxycholate, 0.1% SDS, 150 mM NaCl, 20 mM Tris-HCl, pH 7.5, 2mM EDTA, 50 mM sodium fluoride) containing 10mM N-ethyl maleimide, protease inhibitor cocktail (Roche) and RNase Inhibitor (Promega), and were disrupted by 10 passages through a 25-gauge needle. Cells were centrifuged at 13,000 r.p.m. for 30 min at 4°C and the supernatants were incubated with anti-p53 antibody for 14 h at 4°C with gentle rotation, followed by gentle rotation with 20 μL Protein G Dynabeads (Invitrogen) for 1 h. The beads were washed thrice with Ubi Wash buffer (1% Triton X-100, 50 mM HEPES pH7.5, 150 mM NaCl, 5 mM EDTA, 0.05% SDS) containing 10mM N-ethyl maleimide, protease inhibitor cocktail (Roche) and RNase Inhibitor (Promega), and then twice with PBS containing 10mM N-ethyl maleimide, protease inhibitors and RNase Inhibitor (Promega). After washing, proteins were eluted with 1× SDS sample buffer (50 mM Tris-HCl pH 6.8, 10% glycerol, 2% SDS, 6% β-mercaptoethanol, 0.05% bromophenol blue) containing a protease inhibitor cocktail (Roche). Immunocomplexes were analyzed by SDS-PAGE and immunoblotting with anti-p53 or anti-Ubiquitin antibodies.

**Sequence data analysis**

For RNA-seq analyses of CCSC#P and CCSC#11 cells, 36-bp of single-end reads were sequenced on an Illumina Genome Analyzer IIx as described previously (2) (DRA004047) and raw reads were mapped to the human reference genome (hg19) using TopHat 2.0.8 (<http://tophat.cbcb.umd.edu/>). Gene expression levels were calculated by Cuffdiff 2 (http://cufflinks.cbcb.umd.edu/) on the alignments from TopHat 2.0.8. Additional information such as gene symbols and mRNA names were annotated according to fRNADB databases (4). Genes with alternative splice variants were removed. ncRNAs with *P*-value < 0.05 and upregulated > 1.7-fold (log2) in CCSC#P cells were taken as differentially expressed genes.

RNA-seq samples from HCT116 cells transfected with siRNA targeting *ZNNT1* were sequenced using the Illumina Hiseq 2000 and raw reads were mapped to the human reference genome (hg19) using TopHat 2.0.8. Gene-expression levels were calculated by Cuffdiff 2. Additional information such as gene symbols and mRNA names were annotated according to GENCODE v18 (hg19). Genes with fewer than one fragment per kilobase of exon per million reads mapped were removed. Genes with up- or down-regulated > 2-fold by *ZNNT1* knockdown were taken as differentially expressed genes. “Upstream Regulator” and “Molecular and Cellular Functions” analyses were performed using the Ingenuity Pathway Analysis software tool (IPA; Ingenuity Systems).

**Statistical analysis**

Statistical analysis was performed using a paired (qRT–PCR, RIP) or an unpaired (tumorigenesis, Cell Titer-Glo) two-tailed Student’s *t*-test. A *P*-value < 0.05 was considered to be statistically significant.

**SI References**

1 Taniue K, *et al.* 2020. UHRF1-KAT7-mediated regulation of TUSC3 expression via histone methylation/acetylation is critical for the proliferation of colon cancer cells. *Oncogene*. 39: 1018–1030.

2 Taniue K, *et al.* 2016. Long noncoding RNA UPAT promotes colon tumorigenesis by inhibiting degradation of UHRF1. *Proc Natl Acad Sci U S A*. 113: 1273–8.

3 Taniue K, *et al.* 2016. ASBEL –TCF3 complex is required for the tumorigenicity of colorectal cancer cells. *Proc Natl Acad Sci*. 113: 201605938.

4 Kin T, *et al.* 2007. fRNAdb: a platform for mining/annotating functional RNA candidates from non-coding RNA sequences. *Nucleic Acids Res*. 35: D145-8.


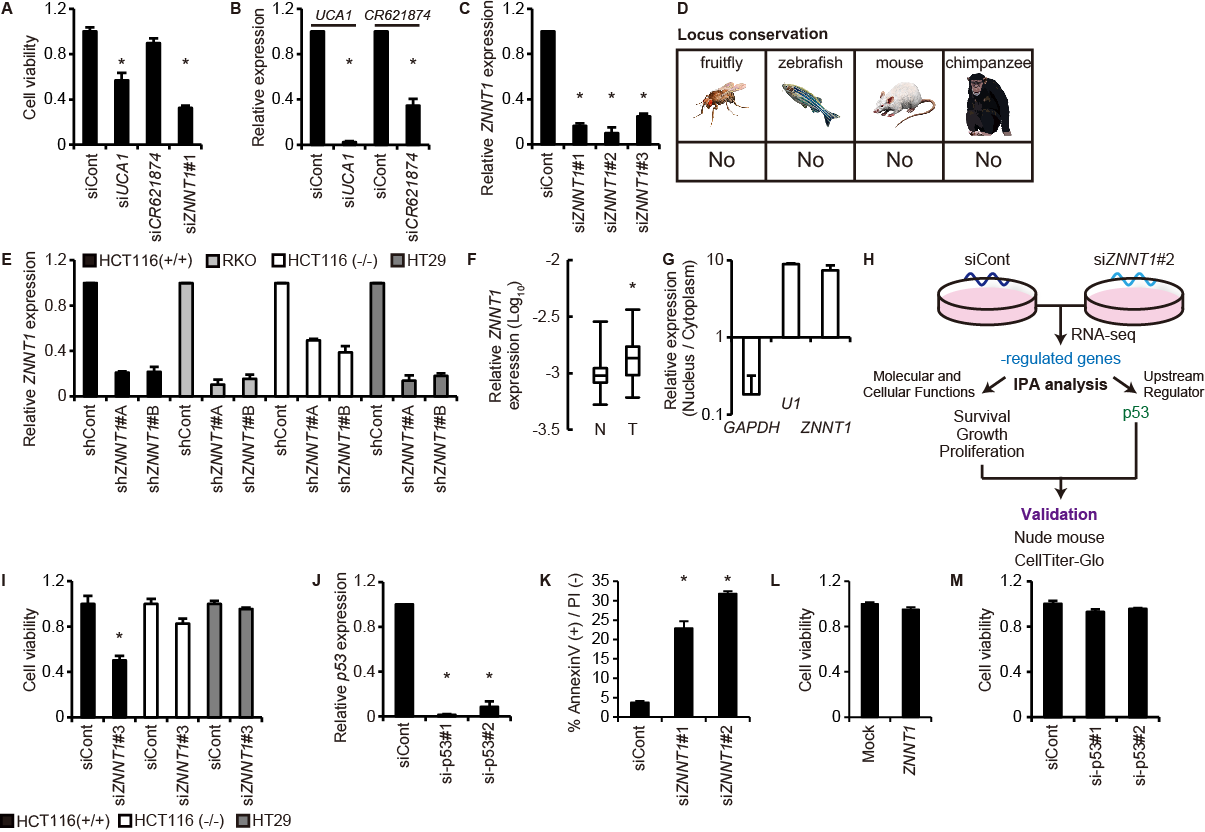
**Fig. S1.** *ZNNT1* is required for the tumorigenicity of colon cancer cells with wild-type p53. (A) Viability of HCT116 cells transfected with an siRNA targeting *UCA1*, *CR621874* and *ZNNT1* was assessed by CellTiter-Glo assays. Results are expressed as the mean ± s.d. (n = 4). **P* < 0.05. (B) qRT-PCR analysis of *UCA1* and *CR621874* expression in HCT116 cells transfected with an siRNA targeting indicated genes. Results are expressed as the mean ± s.d. (n = 3). **P* < 0.05. (C) qRT-PCR analysis of *ZNNT1* expression in HCT116 cells transfected with an siRNA targeting *ZNNT1.* Results are expressed as the mean ± s.d. (n = 3). **P* < 0.05. (D) *ZNNT1* is not conserved in other vertebrates. (E) qRT-PCR analysis of *ZNNT1* expression in HCT116(p53+/+), RKO, HCT116(p53-/-) and HT29 cells infected with a lentivirus harboring an shRNA targeting *ZNNT1*. Results are expressed as the mean ± s.d. (n = 3). **P* < 0.05. (F) qRT-PCR analysis of *ZNNT1* expression in human colon cancerous and corresponding noncancerous tissues (n = 24 pairs). T, tumor tissues. N, noncancerous tissues. **P* < 0.05. (G) Subcellular localization analysis of *ZNNT1*. RNAs were isolated from the nuclear and cytoplasmic fractions of HCT116 cells and quantified by qRT-PCR (n = 3). *GAPDH*, cytoplasmic control; *U1*, nuclear control. (H) Schematic representation of IPA analysis. (I) Viability of HCT116 (p53+/+), HCT116 (p53-/-) and HT29 cells transfected with an siRNA targeting *ZNNT1* was assessed by CellTiter-Glo assays. Results are expressed as the mean ± s.d. (n = 4). **P* < 0.05. (J) qRT-PCR analysis of *p53* expression in HCT116 cells transfected with an siRNA targeting p53*.* Results are expressed as the mean ± s.d. (n = 3). **P* < 0.05. (K) Annexin V assays were performed with HCT116 cells transfected with siRNA targeting *ZNNT1*. Results are expressed as the mean ± s.d. (n = 3). **P* < 0.05. (L) Viability of HCT116 cells transfected with *ZNNT1* was assessed by CellTiter-Glo assays. Results are expressed as the mean ± s.d. (n = 4). (M) Viability of HCT116 cells transfected with an siRNA targeting p53 was assessed by CellTiter-Glo assays. Results are expressed as the mean ± s.d. (n = 4).


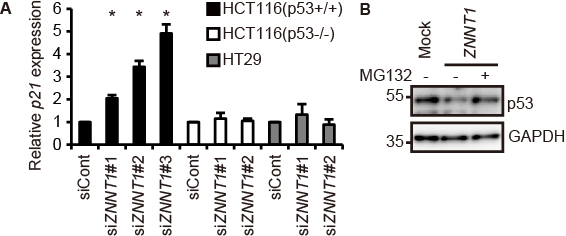
**Fig. S2.** Knockdown of *ZNNT1* inhibits the degradation and ubiquitination of p53 protein in colon cancer cells. (A) qRT-PCR analysis of *p21* expression in HCT116 (p53+/+), HCT116 (p53-/-) and HT29 cells transfected with an siRNA targeting *ZNNT1*. Results are expressed as the mean ± s.d. (n = 3). **P* < 0.05. (B) HCT116 cells transfected with *ZNNT1* were cultured in the presence or absence of MG132 and then subjected to immunoblotting analysis with anti-p53 or anti-GAPDH antibody. GAPDH was used as a loading control.


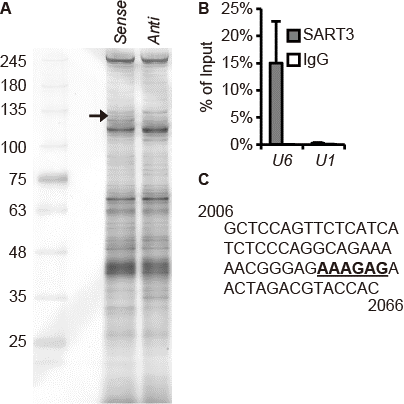
**Fig. S3.** *ZNNT1* is associated with SART3 in colon cancer cells. (A) Biotinylated sense, antisense or mutant *ZNNT1-0* was incubated with lysates from HCT116 cells and then precipitated with streptavidin beads followed by SDS-PAGE and silver staining. The band indicated by the arrowhead was excised and subjected to liquid chromatography-mass spectrometry. (B) Lysates from HCT116 cells were subjected to immunoprecipitation with anti-SART3 antibody or rabbit IgG followed by qRT-PCR analysis to detect *U6* and *U1*. *U6* and *U1* were used as a positive and a negative control, respectively. Results are expressed as the mean ± s.d. (n = 2). (C) Nucleotide sequence of *ZNNT1*-4-2-2. The region to which *ZNNT1* may bind is underlined.


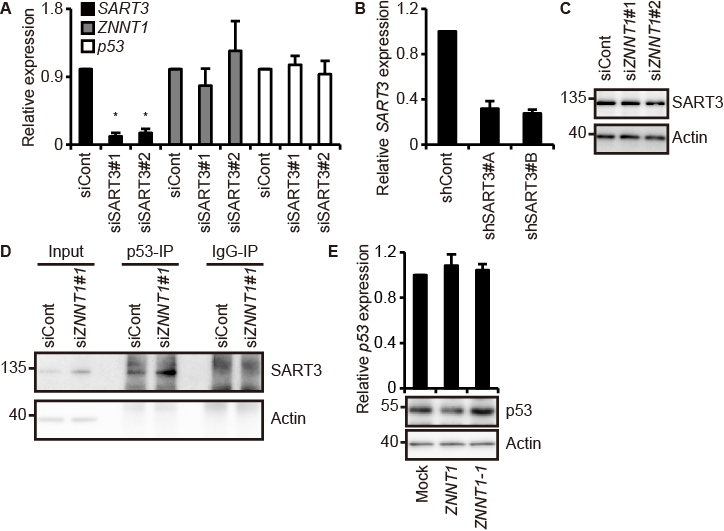
**Fig. S4.** *ZNNT1* regulates the stabilization of p53 by interfering with the binding of SART3 to p53. (A) qRT-PCR analysis of *SART3*, *ZNNT1* and *p53* expression in HCT116(p53+/+) cells transfected with siRNA targeting SART3. Results are expressed as the mean ± s.d. (n = 3). **P* < 0.05. (B) qRT-PCR analysis of *SART3* expression in HCT116 cells infected with a lentivirus harboring an shRNA targeting SART3. Results are expressed as the mean ± s.d. (n = 2). (C) Lysates from HCT116 cells transfected with an siRNA targeting *ZNNT1* were subjected to immunoblotting analysis with anti-SART3 and anti-actin antibodies. Actin was used as a negative control. (D) Lysates from HCT116 cells transfected siZNNT1 (siZNNT1#1) were subjected to immunoprecipitated with anti-p53 antibody or mouse IgG and followed by immunoblotting analysis with anti-SART3 and anti-actin antibodies. (E) (Upper) qRT-PCR analysis of *p53* expression in HCT116 cells transfected with *ZNNT1* or *ZNNT1-1*. Results are expressed as the mean ± s.d. (n = 3). (Lower) Cell lysates were subjected to immunoblotting analysis with anti-p53 or anti-actin antibody. Actin was used as a loading control.


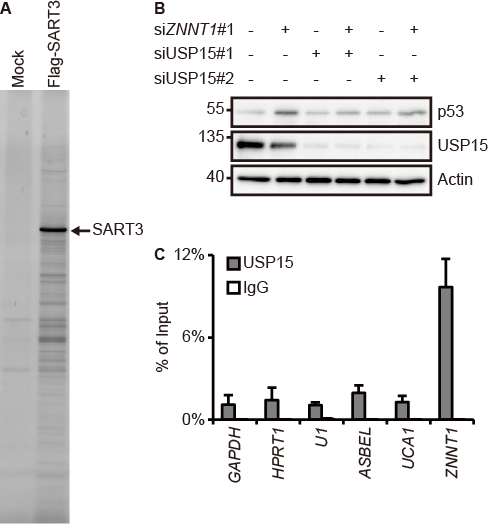
**Fig. S5.** *ZNNT1* regulates stabilization of p53 by interfering with its binding to the SART3-USP15 complex. (A) Lysates from HCT116 cells transfected with Flag-tagged SART3 were subjected to immunoprecipitation with anti-Flag antibody and resolved by SDS-PAGE followed by silver staining. The arrowhead indicates SART3. The immunoprecipitates were subjected to liquid chromatography-mass spectrometry. (B) Lysates from HCT116 cells transfected with siRNA targeting *ZNNT1* (siZNNT1#1) and/or USP15 were subjected to immunoblotting analysis with anti-p53, anti-USP15, and anti-actin antibodies. Actin was used as a negative control. (C) Lysates from HCT116 cells were subjected to immunoprecipitation with anti-USP15 antibody or mouse IgG followed by qRT-PCR analysis to detect *ZNNT1* mRNA. *GAPDH* mRNA, *HPRT1* mRNA, *U1* small nuclear RNA, *ASBEL* and *UCA1* were used as negative controls. Results are expressed as the mean ± s.d. (n = 2).
